# The Bub1-TPR Domain Interacts Directly with Mad3 to Generate Robust Spindle Checkpoint Arrest

**DOI:** 10.1016/j.cub.2019.06.011

**Published:** 2019-07-22

**Authors:** Ioanna Leontiou, Nitobe London, Karen M. May, Yingrui Ma, Lucile Grzesiak, Bethan Medina-Pritchard, Priya Amin, A. Arockia Jeyaprakash, Sue Biggins, Kevin G. Hardwick

**Affiliations:** 1 Institute of Cell Biology, University of Edinburgh, King’s Buildings, Max Born Crescent, Edinburgh EH9 3BF, UK; 2 Howard Hughes Medical Institute, Division of Basic Sciences, Fred Hutchinson Cancer Research Center, Seattle, WA 98109, USA

**Keywords:** Mps1, Bub1, Mad3, spindle checkpoint, mitosis, TPR domain

## Abstract

The spindle checkpoint monitors kinetochore-microtubule interactions and generates a “wait anaphase” delay when any defects are apparent [1, 2, 3]. This provides time for cells to correct chromosome attachment errors and ensure high-fidelity chromosome segregation. Checkpoint signals are generated at unattached chromosomes during mitosis. To activate the checkpoint, Mps1^Mph1^ kinase phosphorylates the kinetochore component KNL1^Spc105/Spc7^ on conserved MELT motifs to recruit Bub3-Bub1 complexes [4, 5, 6] via a direct Bub3 interaction with phospho-MELT motifs [7, 8]. Mps1^Mph1^ then phosphorylates Bub1, which strengthens its interaction with Mad1-Mad2 complexes to produce a signaling platform [9, 10]. The Bub1-Mad1 platform is thought to recruit Mad3, Cdc20, and Mad2 to produce the mitotic checkpoint complex (MCC), which is the diffusible wait anaphase signal [9, 11, 12]. The MCC binds and inhibits the mitotic E3 ubiquitin ligase, known as Cdc20-anaphase promoting complex/cyclosome (APC/C), and stabilizes securin and cyclin to delay anaphase onset [13, 14, 15, 16, 17]. Here we demonstrate, in both budding and fission yeast, that kinetochores and KNL1^Spc105/Spc7^ can be bypassed; simply inducing heterodimers of Mps1^Mph1^ kinase and Bub1 is sufficient to trigger metaphase arrest that is dependent on Mad1, Mad2, and Mad3. We use this to dissect the domains of Bub1 necessary for arrest, highlighting the need for Bub1-CD1, which binds Mad1 [9], and Bub1’s highly conserved N-terminal tetratricopeptide repeat (TPR) domain [18, 19]. We demonstrate that the Bub1 TPR domain is both necessary and sufficient to bind and recruit Mad3. We propose that this brings Mad3 into close proximity to Mad1-Mad2 and Mps1^Mph1^ kinase, enabling efficient generation of MCC complexes.

## Highlights

• Heterodimers of Mps1 and Bub1 generate robust spindle checkpoint arrest in yeasts
• This arrest is independent of kinetochores but requires Bub1-CD1 and the Bub1-TPR
• The Bub1-TPR is both necessary and sufficient for Mad3 interaction and recruitment
• Recombinant fission yeast Bub1-TPR and Mad3 form a stable complex

## Graphical Abstract

**Figure:**
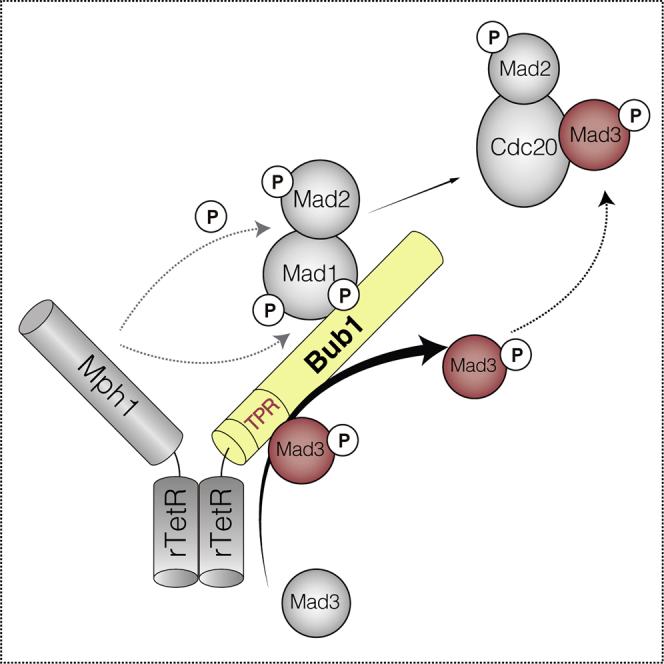

## Teaser

Leontiou et al. demonstrate that heterodimers of Mps1 and Bub1 are sufficient to trigger checkpoint-dependent metaphase arrest in both budding and fission yeast; kinetochores and KNL1 can be bypassed. The *S. pombe* Bub1-TPR domain is both necessary and sufficient to bind and recruit Mad3, enabling efficient generation of MCC complexes.

## Results and Discussion

Forced heterodimerization of Mps1 kinase and Spc105^KNL1^ is sufficient to generate spindle checkpoint arrest in budding yeast [20]. We recently demonstrated that fission yeast cells can be arrested in mitosis by expressing heterodimers of the Mph1^Mps1^ kinase and Spc7^KNL1^ kinetochore protein [21]. However, both studies initiated checkpoint signals from the kinetochore protein Spc105/Spc7/KNL1, and, thus, it could be argued that kinetochore components were still involved, albeit ectopically. This kinetochore component could simply be a passive scaffold upon which checkpoint complexes assemble, but it might also have a role in their activation. KNL1 was initially named because of the “kinetochore null” phenotype after RNA knockdown in *C. elegans* [22]. It has numerous kinetochore-based functions. Although it is a relatively minor microtubule-binding factor, it has been suggested to be part of the “tension sensor” at attached kinetochores [23]; it is the major kinetochore binding site for the Bub3-Bub1 complex in mitosis [4, 5, 6, 8, 10, 24] and the major kinetochore binding site for protein phosphatase 1, which promotes checkpoint silencing [25, 26, 27].

### Rapamycin-Induced Mps1^Mph1^-Bub1 Heterodimers Induce Mitotic Arrest in Budding Yeast Independent of Spc105^KNL1^

**Figure 1:**
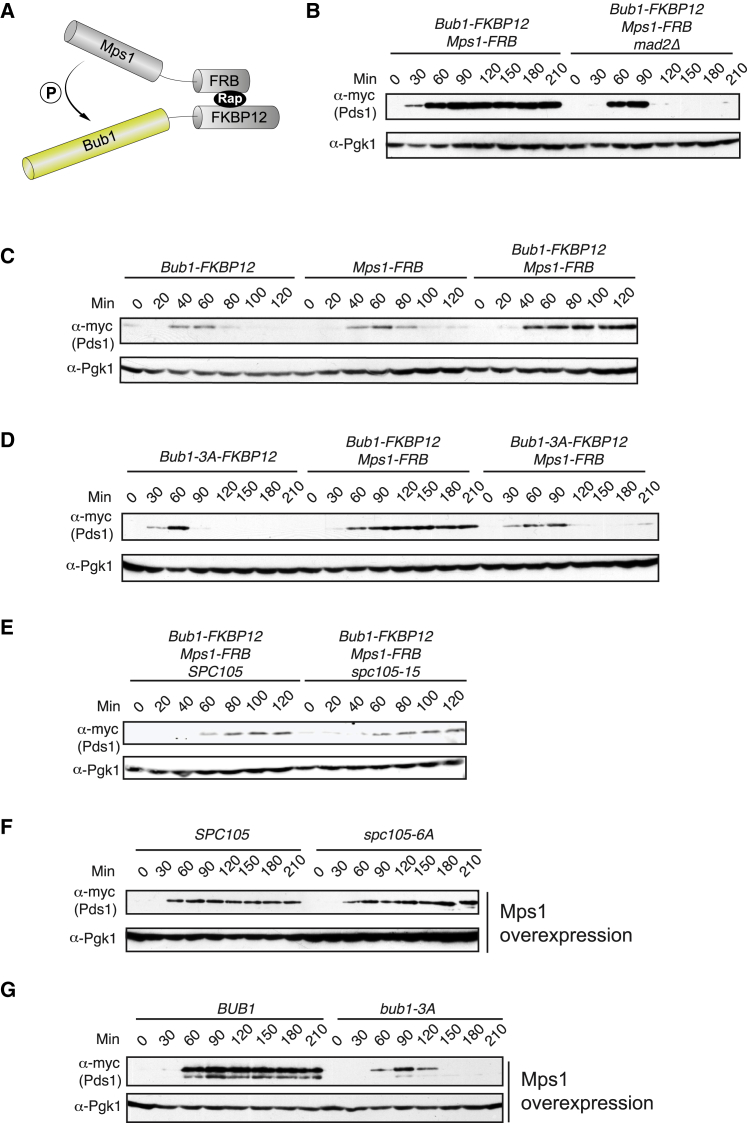
Mps1-Bub1 Anchoring Activates the Checkpoint in Budding Yeast (A) A schematic model of the rapamycin-induced Mps1-Bub1 heterodimer. (B) Strains of the indicated genotype were synchronized in G1 arrest with alpha factor and then released into medium containing 1 μg/mL rapamycin. Pds1 stabilization was monitored, and the strains used were SBY15618 (Bub1-FKBP12 and Mps1-FRB) and a similar strain also lacking Mad2 (SBY15638). Alpha factor was added again approximately 40 min after G1 release. The no-rapamycin control is shown in Figure S1. (C) Pds1 stabilization was monitored as in (B) at 37°C with strains SBY15600 (only Bub1-FKBP12), SBY15659 (only Mps1-FRB), and SBY15618 (both Bub1-FKBP12 and Mps1-FRB). (D) Pds1 stabilization was monitored as in (B) in strains with phospho-deficient Bub1 (SBY15665 [Bub1(3A)-FKBP12], SBY15667 [Bub1(3A)-FKBP12 and Mps1-FRB]) at room temperature. (E) Cells were treated as in (B), except that cells were shifted to 37°C upon alpha factor release to inactivate spc105-15. The strains used were SBY15618 (*SPC105)* and SBY17626 (*spc105-15*). (F) Overexpression of Mps1 kinase (*GAL-MPS1*) arrests budding yeast cells in mitosis, even when the key Spc105 phosphorylation sites are mutated to non-phosphorylatable alanines (*spc105-6A*). Strains SBY12455 and SBY12457 were treated similar as in (B) but were induced with galactose upon alpha factor release. (G) Overexpression of Mps1 kinase (*GAL-MPS1*) does not arrest budding yeast cells in mitosis when the key Bub1 phosphorylation sites are mutated to non-phosphorylatable alanines (*bub1-3A*). Strains SBY15486 and SBY15493 were treated as in (B). See also Figure S1.

Rapamycin can be used to force heterodimerization of two proteins in an inducible fashion. We fused FKBP12 to Bub1 and FKBP12-rapamycin binding (FRB) to Mps1 (Figure 1A) and tested for mitotic arrest in synchronized cells. Figure 1B demonstrates that the combination of these two fusion proteins arrested cells in the presence of rapamycin, with high levels of securin (Pds1) detectable in cell lysates, and that this arrest was both Mad2 dependent and rapamycin dependent (Figure S1A). Thus, heterodimers of Mps1-Bub1 are sufficient to induce a mitotic block in budding yeast. Importantly, neither Bub1-FKBP12 nor Mps1-FRB affected the cell cycle when expressed alone in the presence of rapamycin (Figure 1C). Mad1 recruitment to kinetochores is frequently cited as a major Bub1 checkpoint function [28], so we tested the importance of the Bub1-CD1 domain (using the *bub1-3A* allele [9]). bub1-3A lacks conserved phosphorylation sites that recruit Mad1 to Bub1, and these sites were necessary for rapamycin-induced mitotic arrest (Figure 1D).

Bub1 interacts with kinetochores via Bub3 binding [7] to phosphorylated KNL1^Spc105/Spc7^ [4, 5, 6]. To rule out an accessory role of kinetochore-localized pools of Bub1 in this Mps1-Bub1 arrest, we used the temperature-sensitive *spc105-15* allele, which abrogates kinetochore structure-function and is unable to recruit checkpoint proteins to kinetochores at its restrictive temperature [29]. Figure 1E demonstrates that this mutation did not stop cells expressing Bub1FKBP12-Mps1FRB from arresting upon rapamycin addition. This is consistent with budding yeast [20] and fission yeast studies [21], where kinetochore localization of the Mph1^Mps1^-Spc7^KNL1^ signaling scaffold is not important; arrests were generated independent of kinetochore, spindle pole, and nuclear envelope enrichment. Figure S1B confirms that this *spc105-15* mutation did abrogate nocodazole-induced checkpoint arrest, where unattached kinetochores generate the mitotic checkpoint complex (MCC). These budding yeast experiments lead to a model in which Mps1 kinase first phosphorylates Spc105 to produce a binding site for Bub3-Bub1 complexes and then phosphorylates Bub1 to recruit Mad1-2 complexes. Significant overexpression of Mps1 kinase has long been known to be sufficient to checkpoint-arrest yeast cells [30]. Figure 1F shows that *GAL-MPS1* bypasses the need for Spc105 phosphorylation for either checkpoint activation or Bub1-Mad1 complex formation (Figure S1C), but, importantly, it did not bypass Bub1 phosphorylation (Figures 1G and S1D). This argues that formation of the Bub1-Mad1 complex remains critical, even when cells contain very high levels of active Mps1 kinase and its other checkpoint substrates are likely to be fully modified. A Bub1-Mad1 complex is formed when *GAL-MPS1* is induced in *spc105-6A* cells (Figure S1C) but cannot be formed in *bub1-3A* cells where key Bub1 phospho-sites are mutated (Figure S1D). These data support models in which the Bub1-Mad1 complex forms the key platform for catalytic generation of the MCC, whereas Spc105^KNL1^ primarily acts as a scaffold to localize these proteins at kinetochores with Mps1.

### Co-tethering of Mph1^Mps1^ and Bub1 Is Sufficient to Generate Mitotic Arrest in Fission Yeast

**Figure 2:**
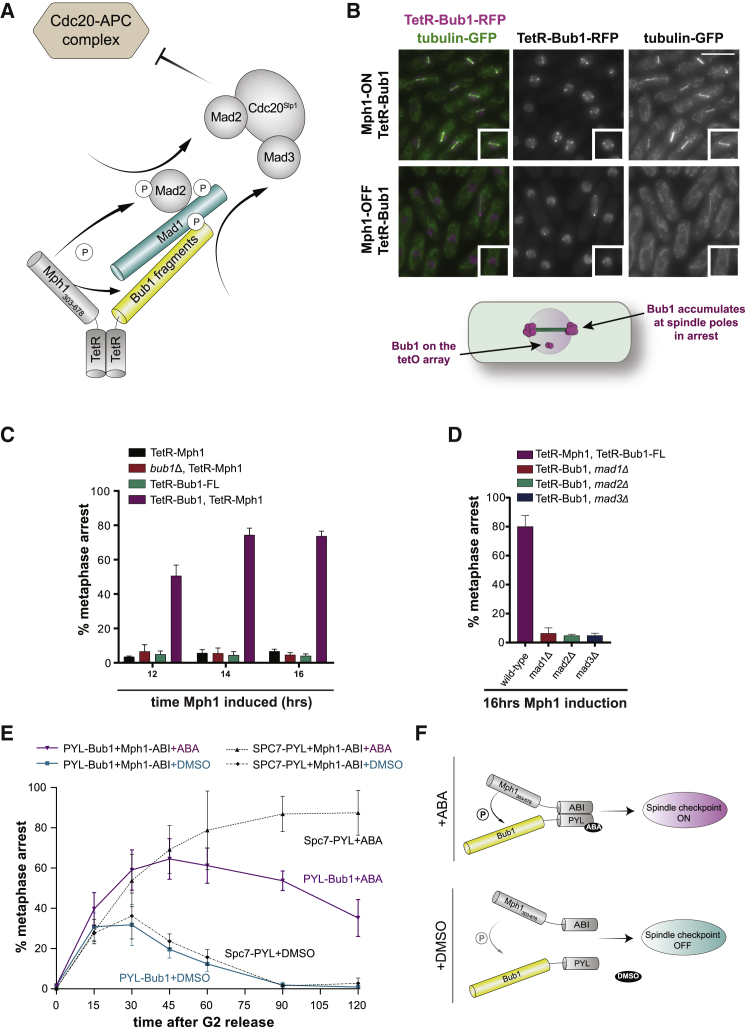
Mph1^Mps1^-Bub1 Dimers Arrest Fission Yeast in Mitosis (A) Schematic model of SynCheck (synthetic checkpoint) arrest driven by heterodimers of TetR-Mph1 and TetR-Bub1. This scaffold recruits Mad1-Mad2 to catalyze production of the MCC (mitotic checkpoint complex; Cdc20-Mad2-Mad3), which then inhibits Cdc20-APC/C. (B) Co-tethering of TetR-Mph1_(303–678)_ with TetR-Bub1 generates robust mitotic arrest with short metaphase spindles. Scale bar, 10 μm. Shown is a schematic of arrested cells. Arrested cells exhibit short metaphase spindles, and Bub1-RFP accumulates on the tetO array and at spindle poles. (C) Quantitation of arrested cells after 12, 14, and 16 h of Mph1^Mps1^ induction; only the strain expressing both TetR-Mph1_(303-678)_ and TetR-Bub1 arrested with short spindles. Thus, expression of either TetR-Bub1FL or TetR-Mph1_(303–678)_ alone is not sufficient for robust arrest. Cells were grown in minimal medium without thiamine to induce the nmt81 promoter. The plus-thiamine control (Mph1^Mps1^ OFF) culture does not arrest, containing just a few mitotic cells. More than 200 cells were analyzed per strain at each time point. The experiments were repeated at least 3 times, and data points are plotted as the mean ± SD. (D) The mitotic arrest is Mad1, Mad2, and Mad3 dependent but independent of endogenous Bub1. The arrest was scored using Atb2-GFP, and more than 200 cells were analyzed per strain at each time point. These strains were analyzed at least three times, and data were plotted as the mean ± SD. (E) Quantification of cultures (with or without abscisic acid [ABA] addition) through a 2-h time course after release from G2. Samples were fixed every 15 min and scored as metaphase arrested when they had short metaphase spindles and a single mass of condensed chromatin. More than 200 cells were analyzed per strain at each time point. The experiment was repeated at least three times, and data were plotted as the mean ± SD. (F) Schematic models of a SynCheck ABA arrest driven by heterodimers of Mph1 and Bub1 induced by ABA addition. See also Figure S2.

We have previously demonstrated that heterodimers of TetR-Spc7^KNL1^ and TetR-Mph1^Mps1^ arrest fission yeast cells in mitosis and generate Bub1-Mad1 complexes [21]. To bypass the need for the fission yeast Spc7^KNL1^ scaffold, we co-expressed TetR-Mph1^Mps1^ with TetR-Bub1 and analyzed the cell-cycle response. The TetR-mCherry-FLAG-Bub1 fusion was expressed constitutively from the adh15 promoter and TetR-FLAG-Mph1^Mps1^ from the weak, thiamine-repressible nmt81 promoter. These yeast cells also contain an *E. coli* tet operator sequence (tetO) array on chromosome 1. The Mph1 fusion lacks its N terminus, which would otherwise target the fusion protein to endogenous kinetochores (Figure 2A). Figure 2B demonstrates robust arrest from the combined effect of expressing TetR-Mph1 and TetR-Bub1, after 12–16 h of nmt promoter induction, in medium lacking thiamine. Importantly, neither construct on its own was sufficient for arrest (Figures 2C, S2C, and S2D), which is consistent with models where phosphorylated Bub1 is a critical signaling output. Figure S2B demonstrates that, although heterodimers of Mps1-Bub1 are necessary, they do not need to be enriched on a tetO array. Arrests were observed with or without anhydro-tetracycline (Figure S2B) and with or without the tetO array (Figure S2B), but, importantly, they were not observed when the TetR domain was removed from the Mph1 fusion protein (Figure S2A). This TetR-based synthetic checkpoint (SynCheck) will likely generate a mixture of homodimers (Mph1-Mph1 and Bub1-Bub1) as well as heterodimers (Mph1-Bub1), and TetR-dimerization is constitutive. To confirm that Mph1-Bub1 heterodimers are what drives this arrest, we employed a chemically induced dimerization system where a complex of two different proteins is only formed in the presence of abscisic acid [31, 32]. Figures 2E and 2F demonstrate that Mph1-ABI and PYL-Bub1 generate robust arrest in the presence of abscisic acid. Here strains were pre-synchronized in G2 using *cdc25* and then released into synchronous mitosis in the presence of abscisic acid or DMSO as a control. Cells expressing Mph1-ABI and PYL-Bub1 maintain short metaphase spindles approximately 90 min longer than control cells, although not as long as our Mph1-Spc7 control. This strongly supports the budding yeast data in Figure 1, where heterodimers of Mps1 and Bub1 are sufficient for checkpoint arrest. We conclude that formation of an Mph1^Mps1^-Bub1 heterodimer is sufficient to induce metaphase arrest in fission yeast.

To test whether this Mph1^Mps1^-Bub1 arrest was dependent on downstream checkpoint proteins, the experiments were repeated in *mad1Δ*, *mad2Δ*, and *mad3Δ* strains (Figure 2D). As was the case for Mps1-KNL1^Spc7^ arrest [21], we found that the Mad1, Mad2, and Mad3 proteins were all required for Mph1^Mps1^-Bub1 arrest. We previously observed spindle pole localization of checkpoint proteins (Bub1, Mad1, Mad2, and Mad3) in SynCheck Mph1^Mps1^-Spc7^KNL1^-arrested cells but found that this Cut7 kinesin-dependent localization of Mad1 is not necessary for arrest [21]. Figures S2E and S2F demonstrate that this is also the case here; TetR-Mph1 and TetR-Bub1 co-expression was able to arrest *mad1-ΔCC* cells in which no spindle pole localization (Figure S2E) of either Bub1 or Mad2 was observed because the Cut7 binding site within the N terminus of Mad1 had been deleted [33].

### Dissection of Bub1 Domains Required for SynCheck

**Figure 3:**
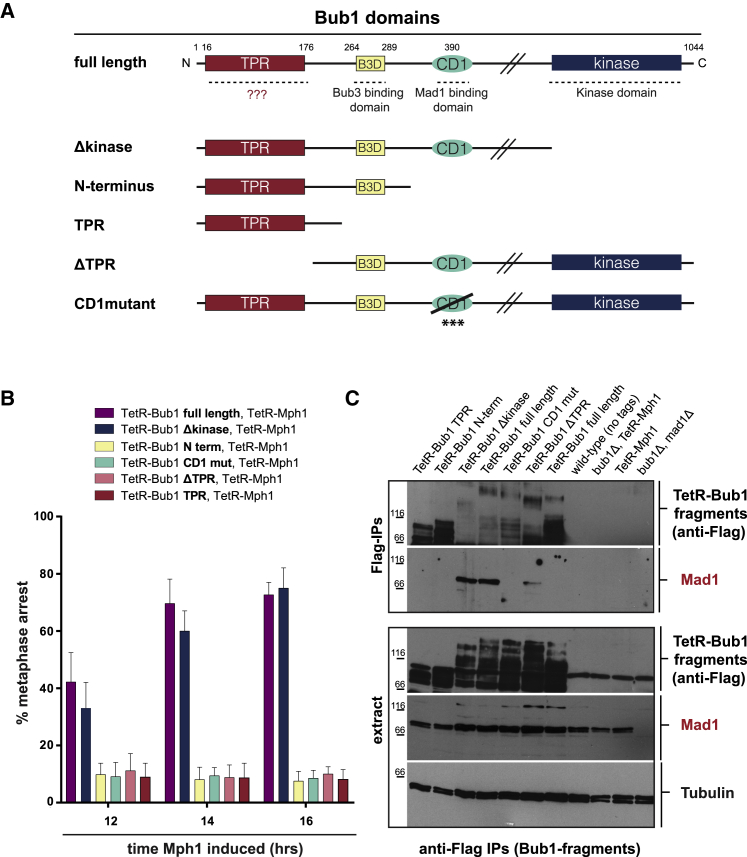
Dissection of Bub1: The TPR and CD1 Domains Are Both Critical for SynCheck Arrest (A) Schematics showing the wild-type plus the six different Bub1 truncations and mutants. ??? indicates the unknown function of the Bub1-TPR domain. The *bub1-CD1* mutation used here was STT-AAA (S381A, T383A, and T386A) [35]. (B) Quantitation of the SynCheck arrests, co-tethering different TetR-Bub1 fragments with TetR-Mph1_(303–678)_. More than 200 cells were analyzed per strain at each time point. This experiment was repeated at least three times, and data were plotted as the mean ± SD. (C) Co-immunoprecipitation (anti-FLAG) and immunoblots (anti-Bub1 and anti-Mad1) demonstrate that a Bub1-Mad1 complex is formed only in the arrested strains (expressing Bub1-full length [FL] or bub1-Δkinase). See also Figure S3.

Bub1 has undergone at least 16 independent occurrences of gene duplication, followed by sub-specialization [34]. In humans, the duplicated genes produce Bub1 and BubR1; in yeast, Bub1 and Mad3. Bub1 is a large polypeptide with several evolutionarily conserved domains [18, 19]. Next we used the Mph1-Bub1 SynCheck assay to test which regions of the Bub1 protein were necessary for arrest. Six different Bub1 fusions were made (Figure 3A): one is full length (FL); one lacks the kinase domain (Δkinase); another also lacks the central domain of Bub1, including CD1 (amino terminus [Nterm]); one lacks the tetratricopeptide repeat (TPR) domain (ΔTPR); one only expresses the N-terminal TPR domain (TPR); and one contains the *bub1*-CD1 mutation (CD1mutant). Log phase cells were washed in –thiamine medium to induce TetR-Mph1 expression and then grown overnight before cytological analysis and quantitation of their arrest. Figure 3B shows that, after 12 h of TetR-Mph1^Mps1^ induction, ∼60% of cells had arrested with short metaphase spindles, and this fraction rose to ∼80% after 16 h. This was true for the FL Bub1 fusion and the one lacking the Bub1 kinase domain, but not the other Bub1 constructs. This experiment demonstrates that Bub1 kinase activity is not necessary for Mph1-Bub1 SynCheck arrest and highlights the importance of the N-terminal TPR domain in addition to CD1. We note that, unlike other domains or motifs that are frequently lost or duplicated during MadBub specialization, the TPR domain is conserved in essentially all Bub1-, BubR1-, and Mad3-related proteins [34].

Phosphorylated Bub1 CD1 recruits Mad1 to kinetochores, and these proteins have been shown to bind directly in budding yeast reconstitution experiments [9]. To test whether Bub1 needs to form a complex with Mad1 in the Mph1^Mps1^-Bub1 SynCheck, we used the *bub1-CD1* mutant, which contains mutations in the conserved region of Bub1 that is phosphorylated and then binds Mad1 [35]. Figure 3 shows that this *bub1-CD1* mutation completely abolished mitotic arrest. We also analyzed complexes formed in Mph1-Bub1 SynCheck arrested cells by immunoprecipitating Bub1 and looking for associated Mad1. Figure 3C shows that, in arrested strains (with Mph1 and either Bub1-FL or Bub1-Δkinase), there were significant levels of the Bub1-Mad1 complex. Figure S3A confirms that the Bub1-Mad1 complex is only formed after thiamine wash-out, Mph1 induction, and mitotic arrest. We conclude that formation of the Bub1-Mad1 complex is a critical step in this fission yeast SynCheck arrest, as in other spindle checkpoint arrests.

### bub3- and spc7-12A Mutants Arrest Well with Mph1^Mps1^-Bub1 Heterodimers

Figure 1D showed that Bub1-Mps1 arrests independent of budding yeast Spc105^KNL1^ function. To confirm this in fission yeast, we carried out two further experiments. Bub3 targets Bub1 to kinetochores, but they only interact with Spc7 after it has been phosphorylated by Mph1 [5, 6, 7]. Mutation of 12 putative phosphorylation sites in Spc7^KNL1^ prevents this interaction with Bub3 complexes [6, 10]. Figures S3B and S3C demonstrate that Bub1-Mph1 arrested efficiently in *spc7-12A* cells. In addition, Bub1-Mph1 arrests in the complete absence of Bub3 (Figures S3B and S3D). We conclude that the Mph1-Bub1 heterodimer arrests independent of KNL1^Spc105/Spc7^ interaction and kinetochore localization in both fission and budding yeast. Interestingly, we see a slight advance in the kinetics of checkpoint arrest in *bub3Δ* (Figure S3C), as one would expect in this assay if Bub3 were acting as an inhibitor of Bub1 in the nucleoplasm, away from kinetochores [6, 10, 21]. Figure S3E demonstrates that foci of checkpoint proteins accumulated on the tetO array and on spindle poles but that they did not co-localize with kinetochores. This is as expected because of the lack of endogenous Mph1 kinase and Bub1 kinase in these strains; Mph1^Mps1^ is critical for the recruitment of all checkpoint proteins to kinetochores in fission yeast [36].

### The N-Terminal Bub1-TPR Domain Is Sufficient to Recruit Mad3

Ideas of how Bub1-like checkpoint proteins are targeted to kinetochores have evolved: initially it was shown that residues 1–331 of the mouse Bub1 protein were sufficient for kinetochore targeting [37], and this was narrowed down to residues 201–300, which contain the Bub3 binding site but lack the TPR domain [38]. Then it was found that human Bub1 proteins could interact directly with motifs in KNL1 (termed KI motifs) via their conserved TPR domains, and it was suggested that this TPR interaction could enhance kinetochore targeting [18, 39, 40, 41]. The human KNL1 KI motif-Bub protein interaction also enhances assembly of KNL1-bound checkpoint complexes [24]. However, these KI motifs are not conserved beyond vertebrates [19]. Bub1 kinetochore interaction in budding yeast, fission yeast, and humans is now thought to be mainly mediated by the Bub3-KNL1 interaction with phosphorylated MELT motifs [7, 8, 10].

How Mad3 gets to fission yeast kinetochores is far from clear. SpMad3 interacts with kinetochores in a Bub1-dependent fashion, but SpMad3 lacks a Bub3 binding domain of its own [36, 42]. SpMad3 also lacks the unstructured domain in hsBubR1, found just after the Bub3-binding domain, which combine to form heterodimers with hsBub1 [43].

**Figure 4:**
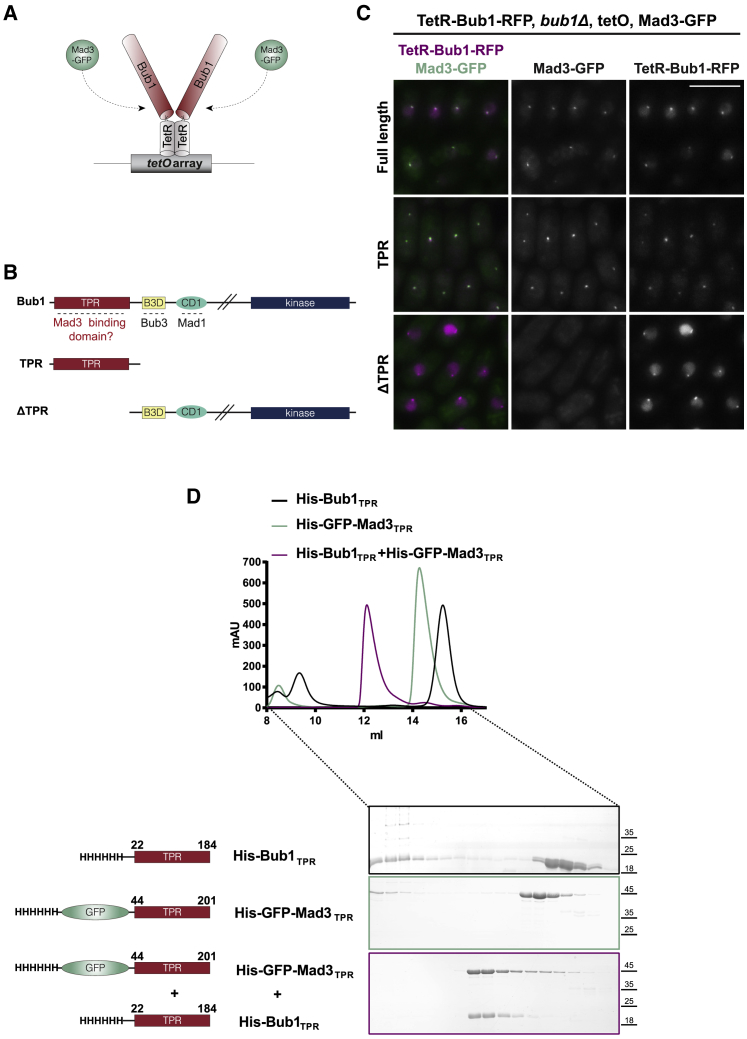
The Bub1-TPR Is Necessary and Sufficient for Mad3 Interaction and Recruitment (A) Schematic model of the TetR-Bub1 tethering assay. Note that it is possible for Bub1 to homodimerize in this system through the TetR domains. (B) Schematics showing the different fragments of *S. pombe* Bub1 fused to TetR and thereby tethered on the tetO array. The two first fragments contain the TPR domain of Bub1 whereas the last one does not. (C) Representative images showing Mad3-GFP and TetR-Bub1-RFP localization. Mad3-GFP co-localized with FL Bub1 and with just the Bub1-TPR but not when the TPR domain was deleted from Bub1. Thus, the Bub1-TPR domain was both necessary and sufficient for Mad3-GFP co-recruitment. Scale bar, 10 μm. See Figure S4 for the corresponding Bub1-Mad3 co-immunoprecipitations. (D) SEC profiles and respective SDS–PAGE analysis of His-Bub1_TPR_ elutes at 15.2 milliliters, His-GFP-Mad3_TPR_ elutes at 14.3 milliliters, and His-Bub1_TPR_/His-GFP-Mad3_TPR_ mix elutes at 12.1 milliliters because of stable Bub1_TPR_-Mad3_TPR_ complex formation. All samples were injected into a Superdex 200 increase 10/300. Absorption at 280 nm (milli absorbance unit [mAU], y axis) is plotted against elution volume (milliliters, x axis). See also Figure S4.

To analyze fission yeast Bub1-Mad3 interactions more directly, we re-purposed our TetR-Bub1 constructs in a tethering assay (Figures 4A and 4B), employing microscopy and strains containing a tetO array. Figure 4C demonstrates that the Bub1-TPR domain is both necessary and sufficient for recruitment of Mad3-GFP to the tetO array. This recruitment is independent of Bub3 because it did not require the Bub3 binding motif in Bub1. Co-immunoprecipitation experiments (Figure S4) demonstrate that Bub1-TPR and Mad3-GFP form a relatively stable complex in these cells. We conclude that an important fission yeast function of the highly conserved Bub1-TPR domain is to recruit and interact with Mad3. To prove that this is a direct interaction, we expressed Bub1-TPR (residues 22–184 aa) and Mad3 (residues 44–201 aa) in bacteria and purified the recombinant proteins. Because they are of similar size, and Mad3 alone is rather insoluble, we fused Mad3 to GFP. Size exclusion chromatography profiles demonstrate that simply mixing the two proteins together *in vitro* was sufficient to produce a stable Bub1-Mad3 TPR complex. Figure S4C demonstrates that this complex formation is driven by TPR-TPR interactions because mixing Bub1-TPR with GFP did not form a complex. Figure 4D also shows that phosphorylation is not needed for formation of the Bub1-TPR-Mad3-TPR complex, although this could still be quite important for its regulation *in vivo*.

### Conclusion

Mps1 kinase is known to have multiple checkpoint targets [11], and its phosphorylation of KNL1 on conserved MELT motifs is necessary to recruit Bub3-Bub1 complexes to kinetochores and initiate checkpoint signaling [7]. Here we bypassed the need for kinetochores and KNL1^Spc105/Spc7^ and showed that phosphorylation of Bub1 by Mps1 is sufficient to initiate checkpoint signaling in both budding and fission yeast. While this manuscript was being revised, a study of synthetic checkpoint signaling in HeLa cells described similar findings [44]. As in all spindle checkpoint arrests, we find that a Bub1-Mad1 complex is formed and necessary for effective downstream signaling [9, 45]. We analyzed the phosphorylation of fission yeast Bub1 and Mad1 by mass spectrometry and found more than 100 sites in Bub1 and more than 10 sites in Mad1 in mitotic cells (data not shown). The complex nature of these modifications makes the design of physiologically relevant phospho-mimic mutants extremely challenging, but this is something we hope to do in future studies.

Another important finding here is that the N-terminal TPR domain of Bub1 is critical for SynCheck arrests. This region of Bub1 is highly conserved through evolution [19, 34] and has been studied in many systems, but its molecular function remains controversial. Mutation of the TPR in yeast leads to strong loss-of-checkpoint-function phenotypes [46, 47]. A similar TPR domain is found in BubR1/Mad3. BubR1 and Mad3 crystal and cryoelectron microscopy (cryo-EM) structures have revealed its importance in forming Mad2 and Cdc20 interactions within MCC-anaphase promoting complex/cyclosome (APC/C) complexes [14, 48]. However, Bub1 does not associate with MCC-APC/C.

As described above, it has been argued that the Bub-TPR domains enhance kinetochore targeting and assembly of checkpoint complexes on the KNL1 scaffold [24, 40, 41]. Our fission yeast experiments bypass both of these functions, suggesting that the key checkpoint effector complexes can be assembled on Bub1 itself when it is sufficiently “primed” by Mps1 phosphorylation. To do this, it needs to bind to both Mad1 (via CD1) and Mad3 (via the TPR).

HsBubR1 interacts with HsBub1 through the Bub3 interaction site and an unstructured domain found just after that [43], but the unstructured region is not conserved in yeast Bub1 and is completely missing in the shorter Mad3 proteins. We have shown here that the TPR region of fission yeast Bub1 is crucial for checkpoint arrest and that it is sufficient to directly interact with and recruit Mad3. We believe that the TPR-mediated interaction between Bub1 and Mad3 is critical for checkpoint signaling in yeast but that it does not matter where this takes place; it can happen on or off kinetochores as long as Mps1 kinase is nearby. The precise role of Bub1-TPR is the subject of ongoing fission yeast studies. Perhaps Bub1 binding simply brings Mad3 close to Mad1-Mad2 in a high local concentration; these complexes are bound to phosphorylated Bub1-CD1. Perhaps Bub1-TPR binding also brings Mad3 close to Mps1 for its efficient phosphorylation [49]. We propose that one or both of these events activate(s) Mad3 for efficient MCC incorporation and APC/C inhibition. Further *in vivo* and *in vitro* experiments will be needed to dissect these possible mechanisms of fission yeast MCC generation.

We also tested recombinant human (Bub1 and BubR1) and *Saccharomyces cerevisiae* (Bub1 and Mad3) TPR domains *in vitro* and found that, unlike the fission yeast domains, they do not form stable heterodimers (size exclusion chromatography [SEC]; data not shown). This is not surprising because the vertebrate Bub proteins have evolved a distinct heterodimerization domain [43], and all four proteins bind directly to Bub3. However, this does not necessarily mean that the fission yeast Bub1-Mad3 interaction is an exception. We are particularly intrigued by a recent analysis of plant Bub1-like proteins, of which there are three [50]. One has a kinase domain, one looks rather like Mad3 and is likely to be part of MCC complexes, and one has three Mad1-binding motifs and, thus, is presumably involved in checkpoint signaling. Surprisingly, none of these proteins appear to have a conserved Bub3-binding domain [34, 50], a property they share with SpMad3. In addition, most plants appear to lack conserved MELT motifs in KNL1 [51]. Dissecting how these plant Bub proteins are targeted to kinetochores and what roles their TPR domains and protein dimerization could have in checkpoint signaling should prove fascinating.

## STAR★Methods

### Key Resources Table

**Table:** 

REAGENT or RESOURCE	SOURCE	IDENTIFIER
**Antibodies**		

Mouse monoclonal anti-FLAG (M2)	Sigma	F3165
Rabbit polyclonal anti-Mad1	Hardwick lab	N/A
Sheep polyclonal anti-GFP	Hardwick lab	N/A
Mouse anti-tubulin	Gull lab	TAT1

**Bacterial Strains**		

ArcticExpress cells	Agilent	Cat #230192

**Chemicals, Peptides, and Recombinant Proteins**		

Abscisic acid (ABA)	Sigma	Cat #A1049
Anhydrotetracycline hydrochloride	Sigma	Cat #37919

**Critical Commercial Assays**		

Gateway cloning	Invitrogen	https://www.invitrogen.com/content/sfs/manuals/gatewayman.pdf
Gibson Assembly	NEB	https://www.neb.com/products/e2611-gibson-assembly-master-mix

**Experimental Models: Organisms/Strains**		

*Saccharomyces cerevisiae* Strain background W303		
*Mata ura3-1 leu2,3-112 his3-11 trp1-1 ade2-1 can1-100 bar1-1 MAD1-3GFP::HIS3 (pSB1601)*	Biggins lab	SBY8416
*Mata ura3-1::pGAL-MPS1-myc::URA3 leu2-3,112 his3-11::pSPC105-SPC105-2V5::HIS3 trp1-1 ade2-1 can1-100 bar1-1 LYS2 spc105Δ::HPH PDS1-18myc::LEU2 (pSB2082)*		SBY12455
*Mata ura3-1::pGAL-MPS1-myc::URA3 leu2-3,112 his3-11::pSPC105-spc105(T149A, T172A, T211A, T235A, T284A, T313A)-2V5::HIS3 trp1-1 ade2-1 can1-100 bar1-1 LYS2 PDS1-18myc::LEU2 spc105Δ::HPH (pSB2083)*		SBY12457
*Mat*α *ura3-1::pGAL-MPS1-myc::URA3 leu2-3,112 his3-11 trp1-1 ade2-1 can1-100 bar1-1 bub1::Bub1-9myc::HIS3 (pSB2420)*		SBY15116
*Mata ura3-1::pGAL-MPS1-myc::URA3 leu2-3,112 his3-11 trp1-1 ade2-1 can1-100 bar1-1 bub1(T485A, T509A, T518A)-9myc::KanMX (pSB1957)*		SBY15237
*Mata ura3-1 leu2,3-112 his3-11 trp1-1 ade2-1 can1-100 bar1-1 SPC105-3FLAG::TRP1 MAD1-3GFP::HIS3 bub1(T485A, T509A, T518A)-9myc::KanMX (pSB1957, pSB1601)*		SBY15310
*Mata ura3-1::pGAL-MPS1-myc::URA3 leu2,3-112 his3-11 trp1-1 ade2-1 can1-100 bar1-1 SPC105-3FLAG::TRP1 MAD1-3GFP::HIS3 bub1(T485A, T509A, T518A)-9myc::KanMX (pSB1957, pSB1601)*		SBY15312
*Mata ura3-1::pGAL-MPS1-myc::URA3 leu2-3,112 his3-11 trp1-1 ade2-1 can1-100 bar1-1 LYS2 BUB1-3V5::KanMX PDS1-18myc:LEU2 (pSB2420)*		SBY15486
*Mata ura3-1::pGAL-MPS1-myc::URA3 leu2-3,112 his3-11 trp1-1 ade2-1 can1-100 bar1-1 LYS2 bub1(T485A, T509A, T518A)-3V5::KanMX PDS1-18myc:LEU2 (pSB2457)*		SBY15493
*Mata ura3-1 leu2-3,112 his3-11:pSpc105-Spc105-2V5:HIS3 trp1-1 ade2-1 can1-100 bar1-1 LYS+ Mad1-3GFP:LEU2 Spc105Δ:HPH Bub1-3FLAG:KanMX (pSB1982, pSB2082)*		SBY15591
*Mata ura3-1 leu2-3,112 his3-11::pSPC105-spc105(T149A, T172A, T211A, T235A, T284A, T313A)-2V5:HIS3 trp1-1 ade2-1 can1-100 bar1-1 LYS2 MAD1-3GFP::LEU2 spc105Δ::HPH BUB1-3FLAG:KanMX (pSB1981, pSB2082)*		SBY15593
*Mata ura3-1::pGAL-MPS1-myc::URA3 leu2-3,112 his3-11::pSPC105-spc105(T149A, T172A, T211A, T235A, T284A, T313A)-2V5::HIS3 trp1-1 ade2-1 can1-100 bar1-1 LYS2 MAD1-3GFP::LEU2 spc105Δ::HPH BUB1-3FLAG::KanMX (pSB1982, pSB2082)*		SBY15594
*Mata ura3-1 leu2-3 his3-11,15 trp1-1 ade2-1 can1-100 bar1-1 tor1-1 fpr1Δ::NAT BUB1-2xFKBP12::HIS3 PDS1-18myc::LEU2*		SBY15600
*Mata ura3-1 leu2-3 his3-11,15 trp1-1 ade2-1 can1-100 bar1-1 tor1-1 fpr1Δ::NAT MPS1-FRB::KAN BUB1-2xFKBP12::HIS3 PDS1-18myc::LEU2*		SBY15618
*Mata ura3-1 leu2,3-112 his3-11 trp1-1 ade2-1 can1-100 bar1-1 MAD1-3GFP::HIS3 BUB1-9myc::KanMX (pSB1601, pSB2420)*		SBY15632
*Mata ura3-1 leu2-3 his3-11,15 trp1-1 ade2-1 can1-100 bar1-1 tor1-1 fpr1Δ::NAT MPS1-FRB::KAN BUB1-2xFKBP12::HIS3 mad2Δ::KanMX PDS1-18myc::LEU2*		SBY15638
*Mata ura3-1 leu2-3 his3-11,15 trp1-1 ade2-1 can1-100 bar1-1 tor1-1 fpr1Δ::NAT MPS1-FRB::KAN PDS1-18myc::LEU2*		SBY15659
*Mata ura3-1 leu2-3 his3-11,15 trp1-1 ade2-1 can1-100 bar1-1 tor1-1 fpr1Δ::NAT bub1(T485A, T509A, T518A)-2xFKBP12::HIS3 PDS1-18myc::LEU2 (pSB2457)*		SBY15665
*Mata ura3-1 leu2-3 his3-11,15 trp1-1 ade2-1 can1-100 bar1-1 tor1-1 fpr1Δ::NAT MPS1-FRB::KAN bub1(T485A, T509A, T518A)-2xFKBP12::HIS3 PDS1-18myc::LEU2 (pSB2457)*		SBY15667
*Mata ura3-1 leu2-3 his3-11,15 trp1-1 ade2-1 can1-100 bar1-1 tor1-1 spc105-15 fpr1Δ::NAT MPS1-FRB::KAN BUB1-2xFKBP12::HIS3 PDS1-18myc::LEU2*		SBY15724
*Mata ura3-1::pGAL-MPS1-myc::URA3 leu2-3,112 his3-11::pSPC105-SPC105-2V5::HIS3 trp1-1 ade2-1 can1-100 bar1-1 LYS2 spc105Δ::HPH Mad1-3GFP::LEU2 BUB1-3FLAG::KanMX (pSB1981, pSB1982)*		SBY15728
*Mata ura3-1 leu2-3 his3-11,15 trp1-1 ade2-1 can1-100 bar1-1 tor1-1 spc105-15 fpr1Δ::NAT MPS1-FRB::KAN BUB1-2xFKBP12::HIS3 PDS1-18Myc::LEU2*		SBY17626
*Mata ura3-1 leu2-3,112 his3-11::pSPC105-spc105(T149A, T172A, T211A, T235A, T284A, T313A)-2V5:HIS3 trp1-1 ade2-1 can1-100 bar1-1 LYS2 MAD1-3GFP::LEU2 spc105Δ::HPH BUB1-3FLAG:KanMX (pSB1981, pSB1982)*	Hardwick lab	NLY1
*Mata ura3-1::pGAL-MPS1-myc::URA3 leu2-3,112 his3-11::pSPC105-spc105(T149A, T172A, T211A, T235A, T284A, T313A)-2V5::HIS3 trp1-1 ade2-1 can1-100 bar1-1 LYS2 MAD1-3GFP::LEU2 spc105Δ::HPH BUB1-3FLAG::KanMX (pSB1981, pSB1982)*	Hardwick lab	NLY2
*Schizosaccharomyces pombe* (Figure 2)		
*lys1::Padh15-rTetR-mCherry-Βub1-FL:ura4 tetO:kanR leu1+:Pnmt81rTetR-mph1*_*(303-678)*_*mph1Δ:natR bub1Δ:hygR GFP-atb2:leu+, mad2-RFP:natR*	Hardwick lab	IL1052
*tetO:kanR leu1+:Pnmt81rTetR-mph1*_*(303-678)*_*mph1Δ:natR bub1Δ:hygR GFP-atb2:leu+, mad2-RFP:natR*		IL1035
*tetO:kanR leu1+:Pnmt81rTetR-mph1*_*(303-678)*_*mph1Δ:natR GFP-atb2:leu+, mad2-RFP:natR*		IL1135
*lys1::Padh15-rTetR-mCherry-Βub1-FL:ura4 tetO:kanR mph1Δ:natR bub1Δ:hygR GFP-atb2:leu+, mad2-RFP:natR*		IL1343
*lys1::Padh15-rTetR-mCherry-Βub1-FL:ura4 tetO:kanR leu1+:Pnmt81rTetR-mph1*_*(303-678)*_*mph1Δ:natR mad3Δ:hygR GFP-atb2:leu+, mad2-RFP:natR*		IL1138
*lys1::Padh15-rTetR-mCherry-Βub1-FL:ura4 tetO:kanR leu1+:Pnmt81rTetR-mph1*_*(303-678)*_*mph1Δ:natR mad1Δ:hygR GFP-atb2:leu+, mad2-RFP:natR*		IL1140
*lys1::Padh15-rTetR-mCherry-Βub1-FL:ura4 tetO:kanR leu1+:Pnmt81rTetR-mph1*_*(303-678)*_*mph1Δ:natR mad2Δ:hygR GFP-atb2:leu+*		IL1142
*Padh41-mph1(303-678)-3xHA-ABI:LEU2 lys1::Padh21-spc7(1-666)- PYL:ura4 cdc25-22 Z:Padh15-mCherry-atb2:natMX6 cdc13-GFP:leu*		PA338
*Padh41-mph1(303-678)-3xHA-ABI:LEU2 lys1::Padh21-PYL-Bub1:ura4 cdc25-22 Z:Padh15-mCherry-atb2:natMX6 bub1Δ:ura4*		IL1624
*Schizosaccharomyces pombe* (Figure 3)		
*lys1::Padh15-rTetR-mCherry-Βub1-FL:ura4 tetO:kanR leu1+:Pnmt81rTetR-mph1*_*(303-678)*_*mph1Δ:natR bub1Δ:hygR GFP-atb2:leu+, mad2-RFP:natR*	Hardwick lab	IL1052
*lys1::Padh15-rTetR-mCherry-Βub1-Δkinase:ura4 tetO:kanR leu1+:Pnmt81rTetR-mph1*_*(303-678)*_*mph1Δ:natR bub1Δ:hygR GFP-atb2:leu+, mad2-RFP:natR*		IL1075
*lys1::Padh15-rTetR-mCherry-Βub1-N terminus:ura4 tetO:kanR leu1+:Pnmt81rTetR-mph1*_*(303-678)*_*mph1Δ:natR bub1Δ:hygR GFP-atb2:leu+, mad2-RFP:natR*		IL1057
*lys1::Padh15-rTetR-mCherry-Βub1-CD1mutant:ura4 tetO:kanR leu1+:Pnmt81rTetR-mph1*_*(303-678)*_*mph1Δ:natR bub1Δ:hygR GFP-atb2:leu+,* mad2-RFP:natR		IL1145
*lys1::Padh15-rTetR-mCherry-Βub1-ΔTPR:ura4 tetO:kanR leu1+:Pnmt81rTetR-mph1*_*(303-678)*_*mph1Δ:natR bub1Δ:hygR GFP-atb2:leu+, mad2-RFP:natR*		IL1260
*lys1::Padh15-rTetR-mCherry-Βub1-TPR:ura4 tetO:kanR leu1+:Pnmt81rTetR-mph1*_*(303-678)*_*mph1Δ:natR bub1Δ:hygR GFP-atb2:leu+, mad2-RFP:natR*		IL1262
*tetO:kanR leu1+:Pnmt81rTetR-mph1*_*(303-678)*_*mph1Δ:natR bub1Δ:hygR GFP-atb2:leu+, mad2-RFP:natR*		IL1035
*tetO:kanR leu1+:Pnmt81rTetR-mph1*_*(303-678)*_*mph1Δ:natR GFP-atb2:leu+, mad2-RFP:natR*		IL1135
*tetO:kanR leu1+:Pnmt81rTetR-mph1*_*(303-678)*_*mph1Δ:natR bub1Δ:hygR mad1Δ:hygR GFP-atb2:leu+, mad2-RFP:natR*		IL1417
*ade6-210 leu1-32 ura4-D18*		KM10
*Schizosaccharomyces pombe* (Figure 4)		
*lys1::Padh15-rTetR-mCherry-Βub1-FL:ura4 tetO:kanR mph1Δ:natR bub1Δ:hygR mad3-GFP:his3*	Hardwick Lab	IL944
*lys1::Padh15-rTetR-mCherry-Βub1-TPR:ura4 tetO:kanR mph1Δ:natR bub1Δ:hygR mad3-GFP:his3*		IL945
*lys1::Padh15-rTetR-mCherry-Βub1-ΔTPR:ura4 tetO:kanR mph1Δ:natR bub1Δ:hygR mad3-GFP:his3*		IL1286
*Schizosaccharomyces pombe* (Figure S2)		
*lys1::Padh15-rTetR-mCherry-Βub1-FL:ura4 tetO:kanR leu1+:Pnmt81-mph1*_*(303-678)*_*mph1Δ::natR mad2-GFP:his3*	Hardwick lab	IL1124
*lys1::Padh15-rTetR-mCherry-Βub1-FL:ura4 tetO:kanR leu1+:Pnmt81rTetR-mph1*_*(303-678)*_*mph1Δ::natR mad2-GFP:his3*		IL724
*lys1::Padh15-rTetR-mCherry-Βub1-FL:ura4 tetO:kanR leu1+:Pnmt81rTetR-mph1*_*(303-678)*_*mph1Δ:natR bub1Δ:hygR GFP-atb2:leu+, mad2-RFP:natR*		IL1052
*lys1::Padh15-rTetR-mCherry-Βub1-FL:ura4 leu1+:Pnmt81rTetR-mph1*_*(303-678)*_*mph1Δ:natR bub1Δ:hygR GFP-atb2:leu+, mad2-RFP:natR*		IL1313
*tetO:kanR leu1+:Pnmt81rTetR-mph1*_*(303-678)*_*mph1Δ:natR bub1Δ:hygR GFP-atb2:leu+, mad2-RFP:natR*		IL1035
*tetO:kanR leu1+:Pnmt81rTetR-mph1*_*(303-678)*_*mph1Δ:natR GFP-atb2:leu+, mad2-RFP:natR*		IL1135
*lys1::Padh15-rTetR-mCherry-Βub1-FL:ura4 tetO:kanR mph1Δ:natR bub1Δ:hygR GFP-atb2:leu+, mad2-RFP:natR*		IL1343
*lys1::Padh15-rTetR-mCherry-Βub1-FL:ura4 tetO:kanR leu1+:Pnmt81rTetR-mph1*_*(303-678)*_*mph1Δ:natR bub1Δ:hygR mad1-ΔCC:hygR GFP-atb2:leu+, mad2-RFP:natR*		IL1501
*ade6-210 leu1-32 ura4-D18*		KM10
*Schizosaccharomyces pombe* (Figure S3)		
*lys1::Padh15-rTetR-mCherry-Βub1-FL:ura4 tetO:kanR leu1+:Pnmt81rTetR-mph1*_*(303-678)*_*mph1Δ:natR bub1Δ:hygR GFP-atb2:leu+, mad2-RFP:natR*	Hardwick lab	IL1052
*lys1::Padh15-rTetR-mCherry-Βub1-Δkinase:ura4 tetO:kanR leu1+:Pnmt81rTetR-mph1*_*(303-678)*_*mph1Δ:natR bub1Δ:hygR GFP-atb2:leu+, mad2-RFP:natR*		IL1075
*lys1::Padh15-rTetR-mCherry-Βub1-CD1mutant:ura4 tetO:kanR leu1+:Pnmt81rTetR-mph1*_*(303-678)*_*mph1Δ:natR bub1Δ:hygR GFP-atb2:leu+,* mad2-RFP:natR		IL1145
*lys1::Padh15-rTetR-mCherry-Βub1-ΔTPR:ura4 tetO:kanR leu1+:Pnmt81rTetR-mph1*_*(303-678)*_*mph1Δ:natR bub1Δ:hygR GFP-atb2:leu+, mad2-RFP:natR*		IL1260
*tetO:kanR leu1+:Pnmt81rTetR-mph1*_*(303-678)*_*mph1Δ:natR bub1Δ:hygR GFP-atb2:leu+, mad2-RFP:natR*		IL1035
*lys1::Padh15-rTetR-mCherry-Βub1-FL:ura4 tetO:kanR mph1Δ:natR bub1Δ:hygR GFP-atb2:leu+, mad2-RFP:natR*		IL1343
*lys1::Padh15-rTetR-mCherry-Βub1-Δkinase:ura4 tetO:kanR leu1+:Pnmt81rTetR-mph1*_*(303-678)*_*mph1Δ:natR bub1Δ:hygR bub3Δ:hygR GFP-atb2:leu+, mad2-RFP:natR*		IL1374
*lys1::Padh15-rTetR-mCherry-Βub1-Δkinase:ura4 tetO:kanR leu1+:Pnmt81rTetR-mph1*_*(303-678)*_*mph1Δ:natR bub1Δ:hygR spc7Δ::ura4+ C::Pspc7-spc7-12A-Tspc7:hygR GFP-atb2:leu+*		IL1594
*lys1::Padh15-rTetR-mCherry-Βub1-Δkinase:ura4 tetO:kanR leu1+:Pnmt81rTetR-mph1*_*(303-678)*_*mph1Δ:natR bub1Δ:hygR spc7Δ::ura4+ C::Pspc7-spc7-WT-Tspc7:hygR GFP-atb2:leu+*		IL1598
*lys1::Padh15-rTetR-mCherry-Βub1- Δkinase:ura4 tetO:kanR mph1Δ:natR bub1Δ:hygR Fta3-GFP:KanR*		IL1106
*Schizosaccharomyces pombe* (Figure S4)		
*tetO:kanR mph1Δ:natR bub1Δ:hygR mad3-GFP:his3+*	Hardwick lab	IL916
*lys1::Padh15-rTetR-mCherry-Βub1-FL:ura4 tetO:kanR mph1Δ:natR bub1Δ:hygR mad3-GFP:his3*		IL944
*lys1::Padh15-rTetR-mCherry-Βub1-TPR:ura4 tetO:kanR mph1Δ:natR bub1Δ:hygR mad3-GFP:his3*		IL945
*lys1::Padh15-rTetR-mCherry-Βub1-ΔTPR:ura4 tetO:kanR mph1Δ:natR bub1Δ:hygR mad3-GFP:his3*		IL1286
*ade6-210 leu1-32 ura4-D18*		KM10

**Recombinant DNA**		

pFA6A-FRB-KanMX6	P30578	Euroscarf
pFA6A-2xFKBP12-HIS3MX6	P30583	Euroscarf

**Software and Algorithms**		

Prism version 7	GraphPad	https://www.graphpad.com/scientific-software/prism/
SlideBook version 5.5	3i	https://www.intelligent-imaging.com/slidebook

### Lead Contact and Materials Availability

Further information and requests for resources and reagents, such as plasmids and yeast strains, should be directed to and will be fulfilled by the Lead Contact, Kevin Hardwick (kevin.hardwick@ed.ac.uk).

### Experimental Model and Subject Details

#### Budding yeast strains and media

Cells were grown in standard YPD media with 2% glucose. For galactose induction, cells were cultured in YEP media with 2% raffinose and induced with 2% galactose. All strains are derivatives of W303. See Key Resources Table for complete strain lists.

#### Fission yeast strains and media

See Key Resources Table for complete strain lists. Cells were grown in standard YES (rich) media at 32°C. For induction of nmt promoter to generate SynCheck arrests, fission yeast cells were first grown on YES plates at 32°C overnight. The following morning, cells were transferred to liquid PMG medium containing with 15 μM thiamine and incubated at 30°C with shaking. After 7 hours, cells were washed 3 times with fresh PMG medium. The cells were then transferred to fresh PMG (without thiamine) containing 10 μM anhydrotetracycline (Sigma, 10mM stock) and incubated at 30°C for 12, 14, 16 and 18hrs.

#### *E. coli* strain and media

ArcticExpress cells (Agilent) were grown in LB broth and induced at 14°C overnight for expression of recombinant TPR proteins.

### Method Details

#### Budding yeast

Bub1-FKBP12 and Mps1-FRB strains were constructed by PCR-based integration of the tags using Euroscarf plasmids P30578 (pFA6A-FRB-KanMX6) and P30583 (pFA6A-2xFKBP12-HIS3MX6). Genomic integrations were verified by marker counter-selection or PCR-based analysis. 3xGFP and 3xFlag strains were constructed by PCR-based integration of tags at the endogenous locus [52], and subsequent backcrossing.

##### Bub1-3A plasmid construction

A BUB1 endogenous replacement construct was generated by inserting Bub1 sequences into a HIS3 integrating vector. First, an existing SPC105 integrating vector pSB1332 [4] was mutagenized with oligonucleotides SB4347-4348 to generate an AgeI restriction site in the vector downstream of HIS3 (pSB2211). The Bub1 3′UTR was next amplified from genomic DNA with SB4749-4750, digested with AgeI, and ligated into this vector to yield pSB2419. Next, pBUB1-BUB1 sequence was digested from existing plasmid pSB1983 with BamHI/XhoI and ligated into pSB2419 to generate pSB2420. Finally, bub1-3a sequence was digested from existing plasmid pSB2055 with XmaI/BamHI and ligated into a Bub1 integrating vector, pSB2420. The resulting plasmid, pSB2457, replaces endogenous Bub1 upon integration. Plasmids were verified by sequencing and restriction digestion analysis at each step.

##### Pds1 time course experiments

Cells were cultured at ambient temperature to OD_600_ of 0.2-0.5 then arrested by alpha-factor treatment (1 ug/mL) for 2-3 hours. G1 arrest was verified by microscopy. Arrested cells were pelleted and resuspended twice in media lacking alpha-factor. Cells were then washed into fresh media to start the time course and rapamycin or nocodazole was added. Media with 2% galactose was used for galactose induction, and 37° media was used to initiate temperature shifts during alpha-factor washout. Fresh alpha-factor was added to cells once rebudding was visible to ensure cells only cycled through mitosis once. At each time point, 1 mL of culture was briefly centrifuged to pellet the cells, which were then snap-frozen in liquid nitrogen. Pellets were resuspended in SDS sample buffer containing PMSF (50 mM Tris pH, 6.8, 2% SDS, 10% glycerol, 1% beta-mercaptoethanol, 0.02% bromphenol blue, 2 mM phenylmethylsulfonylfluoride), lysed by bead beating, and analyzed by western blotting. anti-Pgk1 was purchased from Invitrogen and anti-myc 9E10 was purchased from Covance.

#### Fission yeast – construction of TetR fusion constructs

##### Pnmt81-2xFLAG-rTetR-Mph1_303-678_

The rTetR was amplified out from pAK2 (gift from Alexander Kagansky, Allshire lab), digested with NheI and AseI, and inserted into a pHFF81C vector (gift from Ken Sawin) digested with NheI and NdeI. Mph1_(303-678)_ was amplified from genomic DNA (strain from Silke Hauf) and inserted into tTetR-pHFF81C using Gateway recombination [21].

##### PLYS1U-Padh15-NLS-rTetR-mCherry-2xFLAG-Bub1 fragments

The pRAD15 (gift from Robin Allshire) was amplified using phosphorylated primers before DpnI digestion and re-ligation to re-create the vector with NheI and XhoI sites. These sites were then used to insert a PCR fragment NLS-rTetR-mCherry-FLAG-ccdB into pRAD15. Padh15-NLS-rTetR-mCherry-2xFLAG-ccdB was then amplified out and subsequently joined to a PCR fragment containing the pLYS1U backbone (gift from Jonathan Millar) using KpnI and XhoI to form pLYS1U-Padh15-NLS-rTetR-mCherry-2xFLAG-ccdB (backbone plasmids were constructed by Ivan Yuan [21]). Bub1 fragments (Bub1_FL_, Bub1_Δkinase_, Bub1_TPR_, Bub1_N terminus_, Bub1_CD1mut_ and Bub1_Δ__TPR_) were then inserted into this vector by Gateway recombination. Gateway cloning was performed using kits (LR Clonase II Enzyme Mix, BP Clonase II Enzyme Mix) obtained from Invitrogen in accordance with the manufacturer’s instructions.

##### Construction of the Mad1 N-terminal truncation (mad1-ΔCC)

To truncate Mad1 expressed from its endogenous promoter, 762bp containing the promoter region, 325bp of flanking sequence upstream of this and 566bp of mad1 coding sequence, excluding the first 500bp of mad1, were amplified from genomic DNA. The hygromycin resistance cassette was amplified from pFA6hphMX. The flanking sequence was digested with KpnI and Sal1 and cloned into pBluescript, then the resulting vector was digested with Sal1 and EcoRV and the remaining fragments were assembled by Gibson Assembly (NEB) in the following order; hygR, promoter then coding region, to give *pMad1ΔCC*-hyg. To GFP tag the N terminus of full length Mad1 the endogenous promoter was replaced by Padh21 and GFP sequences inserted before 1kb of mad1WT coding sequence as described above, using Sal1 and EcoRV digested vector and Gibson assembly. The assembled sequences were amplified by PCR and transformed into fission yeast.

#### Construction of Mph1-ABI and Bub1-PYL

##### P_adh41_-mph1_303-678_-3xHA-ABI

Mph1_303-678_ was amplified from a pDONR 201 plasmid containing Mph1_303-678_. 3xHA was amplified from a plasmid from the Allshire lab containing codon optimized PYL-3xHA. ABI was amplified from a pMT_CID_ABI_VS_H vector from the Heun lab. These PCR fragments were Dpn1 treated and assembled into a Sma1-digested and antarctic phosphatase treated, gel purified pRad41 yeast expression vector by Gibson assembly.

##### pLYS1U-Padh21-PYL-Bub1

The yeast expression vector pLYS1U-P_adh21_-NLS-rTetR-mCherry-2xFLAG-Spc7_1-666_ ([21], with a modified adh promoter TATA box: TAAATA for adh21) was digested with Nhe1 and Xho1 and gel purified to isolate the vector backbone pLYS1U. PYL (amplified from the bVNI-221 vector from the Heun laboratory) and Bub1 (amplified from genomic DNA) were then assembled into the digested vector backbone using Gibson assembly.

##### pLYS1U-Padh21-NLS-Spc7_1-666_-PYL

The yeast expression vector pLYS1U-Padh21-NLS-rTetR-mCherry-2xFLAG-spc7_1-666_ [21] (with a modified adh promoter TATA box: TAAATA for adh21) was digested with Nhe1 and Xho1 and gel purified to isolate the vector backbone. Spc7_1-666_ was amplified from pLYS1U-Padh21-NLS-rTetR-mCherry-2xFLAG-spc7_1-666_ [21] containing wild-type Spc7. PYL was amplified from a bVNI-221 vector from the Heun laboratory. The fragments were then assembled into the digested vector backbone using Gibson Assembly. A Not1 digest linearized the plasmid for yeast integration.

##### Construction of the His-Bub1_TPR_ and His-GFP-Mad3_TPR_

Bub1_TPR_ truncation was amplified from “pDONR201-Bub1-AC” by PCR. The PCR products were ligated into pET-DUET using Quick Ligase then 1.5 μL transformed into DH5α *E. coli* according to standard protocol. The Mad3_TPR_ was amplified by PCR and cloned into the 9GFP (N-terminal His-GFP tag) LIC vector. The vector was digested with SspI enzyme and both the vector and the PCR products were run on an agarose gel. The fragments were gel purified and the exonuclease reaction performed using T4 DNA polymerase. The PCR fragments were mixed with the vector and transformed in XL1 Blue *E.coli* cells according to standard protocol. Colonies were screened by PCR and then sequenced. Correct plasmids were then transformed into Arctic cells DE3 to induce expression of the proteins.

##### cdc25-22 synchronization and abscisic acid arrest

Cells were grown at 25°C for 1-2 days on YES (rich yeast media, with additional leucine, arginine, lysine, histidine and uracil) plates. They were then pre-cultured in 10 mL of liquid YES containing amino acid supplements at 25°C over the day and inoculated into a larger culture of YES overnight. The following day, log phase cultures were shifted to 36°C for 3.5 h to block in G2. After this, cultures were cooled quickly in iced water to rapidly shift them back to 25°C and release them from the G2 block. For the synthetic arrest assay following a *cdc25-22* block, 250 mM ABA stock (Sigma Aldrich A1049) was added to cultures 5 min after release to achieve a final concentration of 250 μM (unless otherwise stated). Samples were collected every 15min and fixed with methanol.

##### TetR-induced SynCheck assay

For the synthetic arrest: fission yeast cells were first grown on YES (rich) plates at 32°C overnight. The following morning, cells were transferred to 10 mL of liquid PMG medium containing 15 μM thiamine and were incubated at 30°C with shaking. After seven hours, cells were harvested by spinning at 6,000 RPM for 2.5 min and washed 3 times with fresh PMG (containing supplements). The cells were transferred to fresh PMG (without thiamine) containing 10 μM anhydrotetracycline (Sigma, 10mM stock), and then incubated at 30oC for 12, 14, 16 and 18hrs. Depending on the experiment the next morning the cells were harvested by spinning at 6,000 RPM for 2.5 min. The cells were washed with 1ml of clear PMG (without glucose) and harvested by spinning at 6,000 RPM for 1 min. The supernatant was removed (a small volume of media was left, depending on the pellet size) and 6-10 μL of cells was deposited on a glass slide and covered with a glass coverslip.

##### Fluorescence microscopy

Fixed or live cells were imaged immediately using a 100x oil immersion lens and a Zeiss Axiovert 200M microscope (Carl Zeiss Ltd.), equipped with a CoolSnap CCD camera (Photometrics) and Slidebook 5.0 software (3i, Intelligent Imaging Innovations). Typical acquisition settings: 300 ms exposure (FITC & TRITC), 2x binning, Z series over 3 mm range in 0.5 mm steps (7 planes).

#### Co-immunoprecipitation experiments

Fission yeast cells were grown at 30°C overnight in 1.5 l of PMG. For synthetic arrest experiments, the mitotically arrested cells were harvested after 16hrs of Mph1 induction. The cells were harvested by centrifugation at 4000 rpm at 4°C, for 10 minutes (using a Beckman centrifuge). Pelleted cells were frozen into small sized drops using liquid nitrogen and immediately processed to lysis or stored at - 80°C until further use. The cells were ground manually using a mortar and pestle. Yeast powders were resuspended in lysis buffer containing 50mM HEPES pH7.6, 75mM KCl, 1mM MgCl_2_, 1mM EGTA, 10% Glycerol, 0.1% Triton X-100, 1mM Na_3_VO_4_, 10 μg/mL CLAAPE (protease inhibitor mix containing chymostatin, leupeptin, aprotinin, antipain, pepstatin, E-64 all dissolved in DMSO at a final concentration of 10 mg/mL), 1mM Pefabloc, 0.01mM microcystin). Approximately 1g of powder were resuspended in 1ml of lysis buffer. The cells were lysed by sonication (5 s ON and 5 s OFF for a total of 1 min). After sonication the samples were transferred in 1.5 mL tubes and the cell debris was pelleted using centrifugation (10min, at 13000rpm, at 4°C). The lysate was then incubated with anti-GFP or anti-Flag-coupled Dynabeads (Invitrogen) for 15-20 minutes at 4°C. The beads were washed four times with wash buffer (50mM HEPES pH7.6, 75mM KCl, 1mM MgCl2, 1mM EGTA, 10% Glycerol) and once with PBS+0.0001% Tween 20. The proteins were eluted from the beads by adding 2X sample buffer containing DTT. The samples were incubated with sample buffer at room temperature for 15 minutes, then run on an SDS-PAGE gel.

For budding yeast Co-IPs, cells were grown to OD_600_ of approximately 1 at ambient temperature, then induced with galactose addition. Cells were harvested after 2 hours and frozen in liquid nitrogen. Cells were lysed by bead beating or by freezer milling in Buffer H (25 mM HEPES, pH 8.0, 150 mM KCl, 2 mM MgCl2, 0.1 mM EDTA, 0.5 mM EGTA, 15% glycerol, 0.1% NP40, 2 mM DTT) containing protease inhibitors (0.2 mM PMSF, plus either 10 ug/mL each Leupeptin, Pepstatin, and Chymostatin or protease inhibitor cocktail (Roche # 04693132001) and phosphatase inhibitors (1 mM sodium pyrophosphate, 2 mM sodium β-glycerophosphate, 0.1 mM sodium orthovanadate, 5 mM sodium fluoride, and 0.5 ng/mL microcystin). Lysate was clarified by high-speed centrifugation for 30 min at 4°. Supernatant was collected and immunoprecipitated with anti-Flag (M2, Sigma) or anti-GFP (Living Colors) conjugated Protein G Dynabeads (Invitrogen) at 4° for 3 hours. Beads were then washed 5 times with lysis buffer (omitting inhibitors and DTT after the first three washes), then eluted with SDS sample buffer with 5% β-mercaptoethanol for immunoblot analysis.

#### Bacterial lysis and His-tag protein purification

Cell pellets were thawed on ice and resuspended in lysis buffer containing 50mM Tris pH8.0, 500mM NaCl, 10% glycerol, 10mM imidazole, 5mM β-mercaptoethanol, EDTA-free inhibitor tablet (Roche, 1 tablet per 50ml), 1mM Pefabloc. The cells were lysed by sonication (60% amplitude, 1 s ON and 2 s OFF for a total of 6 min). To remove the cell debris, lysed cells were centrifuged at 20,000 rpm, for 30-45 min, at 4°C. Thereafter, the lysate was filtered through a 0.45 um syringe. Both *S.pombe* His-GFP-Mad3_TPR_ and His-Bub1_TPR_ were purified using immobilised ion metal affinity chromatography (IMAC). Lysates from 5g bacteria were incubated (agitation/rotation) for 30-60 min (at 4°C) with 5 mL of cobalt resin. The beads were pre-equilibrated in lysis buffer lacking protease inhibitors. After incubation, the beads were transferred to a Biorad column and washed with 20 column volumes of wash buffer. Proteins were eluted from the beads using the lysis buffer, without protein inhibitors, containing 250mM imidazole. Peak fractions were dialysed overnight (50mM Tris pH 8.0, 150mM NaCl, 5% glycerol).

#### SEC - Size exclusion chromatography

After dialysis, the protein concentrations were measured using a NanoDrop. The proteins were concentrated using 10kDa cut off Vivaspin concentrators at 4500 rpm at 4°C. Samples were then injected (500 μL of concentrated sample) into a Superdex 200 increase 10/300 (GE Healthcare) equilibrated with 50mM Tris pH 8.0, 150mM NaCl, 5% glycerol and 2mM DTT. The buffer was filtered and the gas was removed prior to use. Fractions were analyzed on SDS–PAGE and stained with Coomassie blue.

### Quantification and Statistical Analysis

SynCheck arrest experiments were repeated at least three times, scoring at least 200 cells per strain for each time-point, and the data plotted as the mean ± SD, using GraphPad Prism software. Details of the number of experimental repeats, number of cells analyzed, and the relevant statistics are detailed in the figure legends.

### Data and Code Availability

This study did not generate or analyze datasets or code.

## Supplemental Information

Document S1. Figures S1–S4

Document S2. Article plus Supplemental Information
