# Supplementary material for: The Bub1-TPR Domain Interacts Directly with Mad3 to Generate Robust Spindle Checkpoint Arrest
Source: Curr Biol. 2019 Jul 22;29(14):2407–2414.e7. doi: 10.1016/j.cub.2019.06.011 (PMC6657678; doi:10.1016/j.cub.2019.06.011)
Supplement: Document S1. Figures S1–S4 [file mmc1.pdf]

Current Biology, Volume *29*

Supplemental Information

The Bub1-TPR Domain Interacts Directly with Mad3

to Generate Robust Spindle Checkpoint Arrest

Ioanna Leontiou, Nitobe London, Karen M. May, Yingrui Ma, Lucile Grzesiak, Bethan Medina-Pritchard, Priya Amin, A. Arockia Jeyaprakash, Sue Biggins, and Kevin G. Hardwick

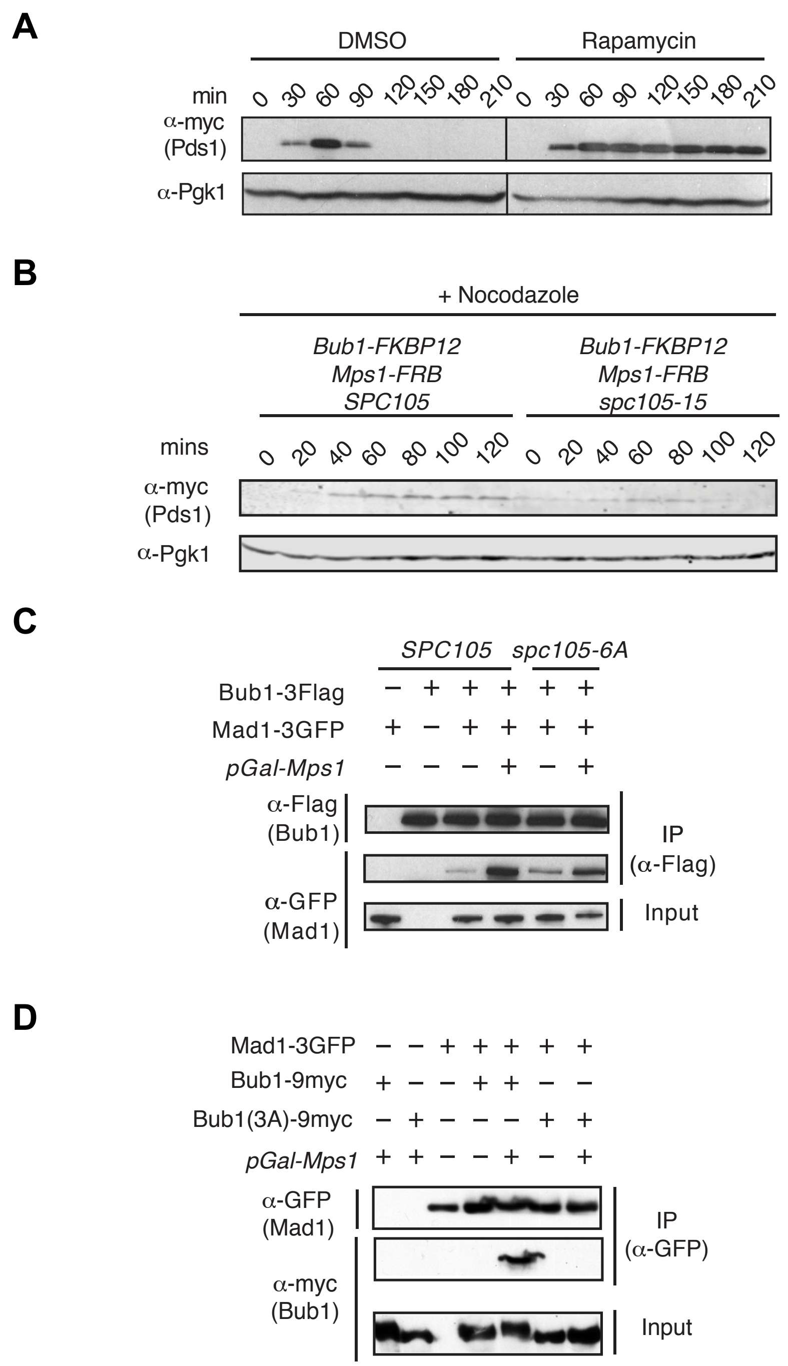

Figure S1. Overexpression of Mps1 bypasses Spc105 but not Bub1 phosphorylation. Related to Figure 1.

A) No rapamycin control. Pds1 was analysed as in Fig. 1B in strain SBY15618, with cells treated with either rapamycin or an equivalent volume of DMSO.
B) The *spc105-15* mutation abrogates the spindle checkpoint arrest induced by nocodazole. Experiment was performed as in Fig. 1E, but using nocodazole (10 ug/mL) in place of rapamycin.
C) A Bub1-Mad1 complex still forms in *spc105-6A* cells when Mps1 kinase is overexpressed (Fig. 1F). Lysates were FLAG-immunoprecipitated from galactose-treated cells and the pulldowns were immunoblotted. Strains used were (left to right) SBY8416, SBY15559, SBY15591, SBY15728, NLY1 and NLY2.
D) A Bub1-Mad1 complex does NOT form in *bub1-3A* cells when Mps1 kinase is overexpressed. Cells were induced with galactose for two hours and lysates were GFP-immunoprecipitated. Strains used were (left to right) SBY15116, 15237, 8416, 15632, 15310, 15312.

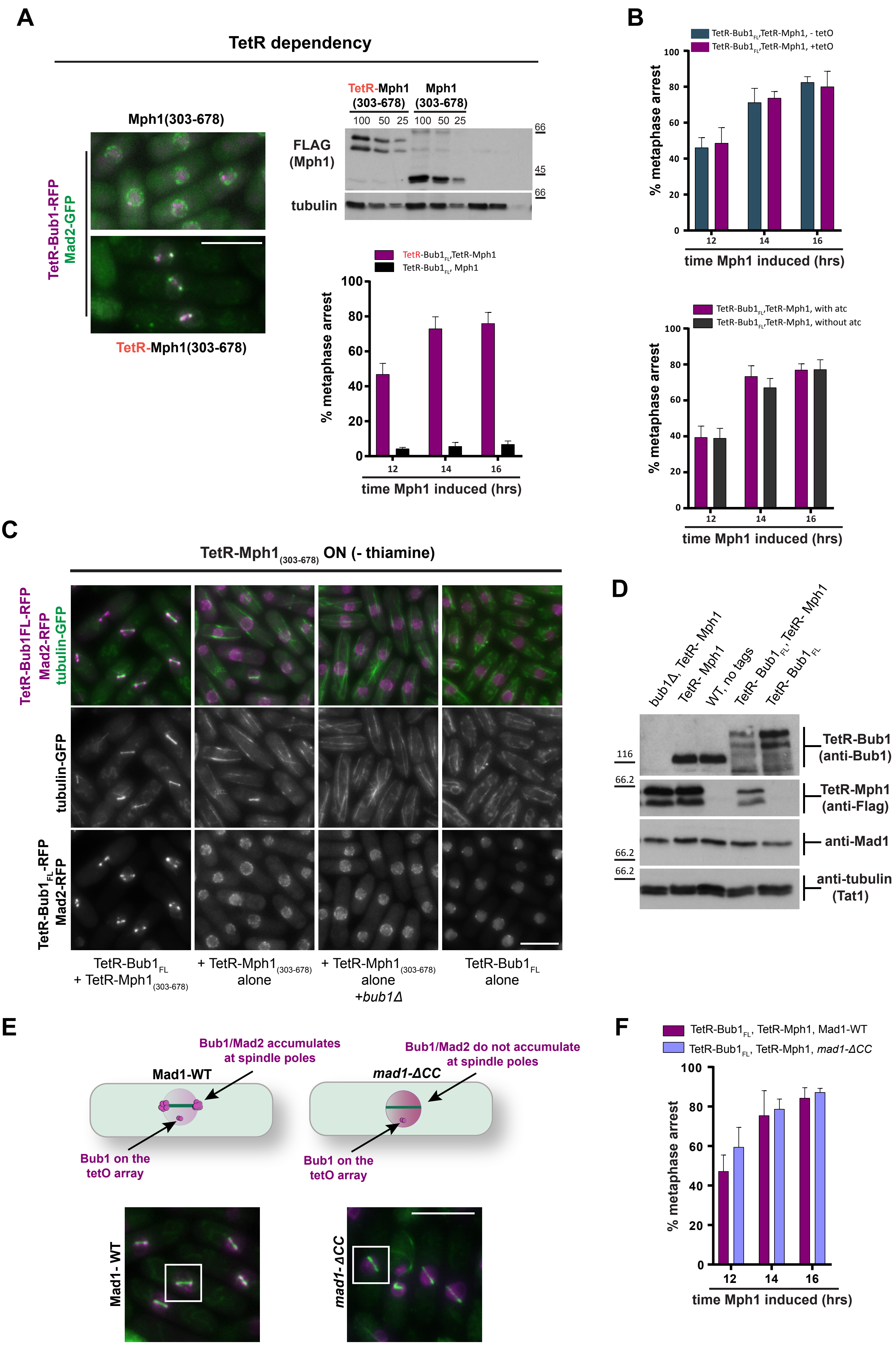
A
TetR dependency
Mph1(303-678)
TetR-Bub1-RFP
Mad2-GFP
TetR-Mph1(303-678)
TetR-Mph1 (303-678)
Mph1 (303-678)
100 50 25 100 50 25
FLAG (Mph1)
tubulin
66
45
66
TetR-Bub1FL,TetR-Mph1
TetR-Bub1FL, Mph1
% metaphase arrest
time Mph1 induced (hrs)
B
TetR-Bub1FL,TetR-Mph1, - tetO
TetR-Bub1FL,TetR-Mph1, +tetO
TetR-Bub1FL,TetR-Mph1, with atc
TetR-Bub1FL,TetR-Mph1, without atc
% metaphase arrest
time Mph1 induced (hrs)
C
TetR-Mph1(303-678) ON (- thiamine)
TetR-Bub1FL-RFP
Mad2-RFP
tubulin-GFP
tubulin-GFP
TetR-Bub1FL-RFP
Mad2-RFP
TetR-Bub1FL + TetR-Mph1(303-678)
+ TetR-Mph1(303-678) alone
+ TetR-Mph1(303-678) alone +bub1Δ
TetR-Bub1FL alone
D
bub1Δ, TetR- Mph1
TetR- Mph1
WT, no tags
TetR- Bub1FL, TetR- Mph1
TetR- Bub1FL
116
66.2
66.2
66.2
TetR-Bub1 (anti-Bub1)
TetR-Mph1 (anti-Flag)
anti-Mad1
anti-tubulin (Tat1)
E
Bub1/Mad2 accumulates at spindle poles
Bub1/Mad2 do not accumulate at spindle poles
Mad1-WT
mad1-ΔCC
Bub1 on the tetO array
Bub1 on the tetO array
Mad1- WT
mad1-ΔCC
F
TetR-Bub1FL, TetR-Mph1, Mad1-WT
TetR-Bub1FL, TetR-Mph1, mad1-ΔCC
% metaphase arrest
time Mph1 induced (hrs)
12
14
16

Figure S2. TetR dimerization, but not tetO binding, is necessary for arrest. Related to Figure 2.

A) No arrest was observed when TetR was removed from the Mph1^{Mps1} fusion protein (Mad2-GFP did not accumulate at spindle poles). Scale bar, 10 microns. Anti-flag immunoblot of whole cell extracts demonstrates that similar levels of TetR-Mph1(303-678) were expressed with and without TetR. Quantitation of cells blocked in metaphase after induction (for 12, 14 and 16 hrs) of Mph1 with or without fusion to TetR. This experiment was repeated 3 times and is plotted as mean ±SD.

B) The tetO array is not necessary for metaphase arrest. The mitotic arrest generated by co-tethering of TetR-Bub1FL with TetR-Mph1(303-678) was compared in strains containing either 112 x tetO or no tetO. This experiment was repeated 3 times and is plotted as mean ±SD. No significant difference was observed.

Comparison of the ability of TetR-Bub1FL with Mph1(303-678) to arrest cells, with and without the presence of anhydro-tetracyclin (atc) in the growth media. This experiment was repeated four times and data plotted as mean ±SD. Atc inhibits TetR binding to the tetO array in these strains, but no significant difference could be observed on cell cycle arrest.

C) Representative images show that co-tethering of TetR-Bub1FL with TetR-Mph1(303-678) generates a robust mitotic arrest with short metaphase spindles and Mad2-RFP and TetR-Bub1-RFP at spindle pole bodies. Tethering of TetR-Bub1FL or tethered TetR-Mph1(303-678) alone does not generate a mitotic arrest. Instead cycling cells display interphase arrays of microtubules and Mad2-RFP stays at the nuclear periphery. Scale bar 10 microns.

D) Immunoblots demonstrate that both TetR-Mph1(303-678) and TetR-Bub1 were expressed in the relevant strains. The Tat1 antibody recognises tubulin and was used here as a loading control.

E) Schematic demonstration of SynCheck arrest in Mad1-WT and *mad1-ΔCC*. The checkpoint components did not move to the spindle pole bodies in *mad1-ΔCC* strains.
The *mad1-ΔCC* allele still arrests with SynCheck even though localisation of Mad1 and Mad2 to the nuclear periphery and spindle poles is lost. This N-terminal coiled-coil domain also includes the Cut7 kinesin interaction site, thus *mad1-ΔCC* also abolishes the localisation of the spindle checkpoint components to the spindle pole bodies. Scale bar is 10 microns.

F) Quantitation of the Mph1-Bub1 SynCheck arrest in *mad1-ΔCC* mutant arrests. This experiment was repeated three times and data plotted as the mean ±SD.

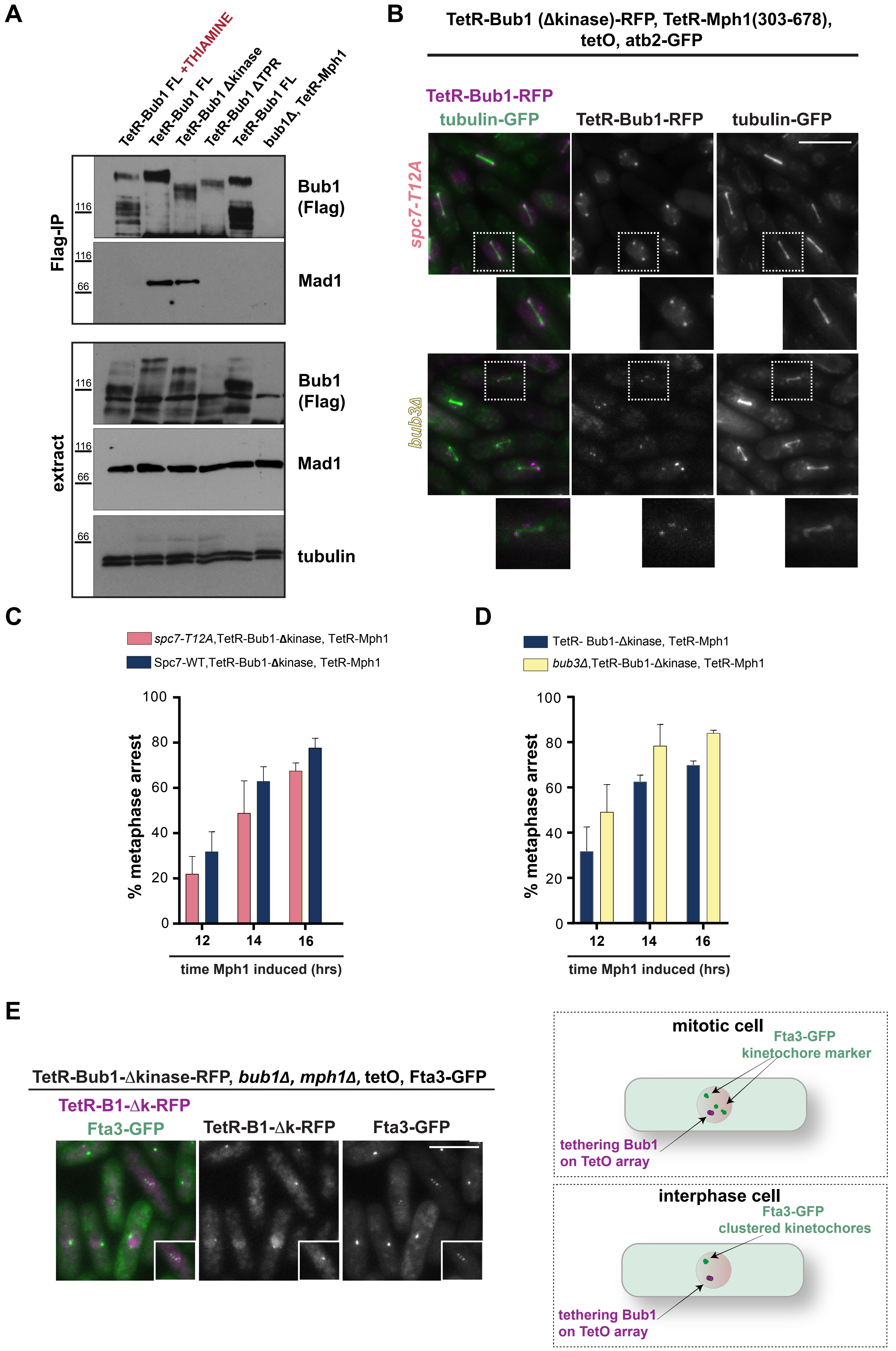
A
TetR-Bub1 FL +THIAMINE
TetR-Bub1 FL
TetR-Bub1 Δkinase
TetR-Bub1 ΔTPR
TetR-Bub1 FL
bub1Δ, TetR-Mph1
Flag-IP
116
116
66
Bub1 (Flag)
Mad1
extract
116
116
66
66
Bub1 (Flag)
Mad1
tubulin
B
TetR-Bub1 (Δkinase)-RFP, TetR-Mph1(303-678), tetO, atb2-GFP
TetR-Bub1-RFP tubulin-GFP
TetR-Bub1-RFP
tubulin-GFP
spc7-T12A
bub3Δ
C
spc7-T12A,TetR-Bub1-Δkinase, TetR-Mph1
Spc7-WT,TetR-Bub1-Δkinase, TetR-Mph1
% metaphase arrest
0
20
40
60
80
100
12
14
16
time Mph1 induced (hrs)
D
TetR- Bub1-Δkinase, TetR-Mph1
bub3Δ,TetR-Bub1-Δkinase, TetR-Mph1
% metaphase arrest
0
20
40
60
80
100
12
14
16
time Mph1 induced (hrs)
E
TetR-Bub1-Δkinase-RFP, bub1Δ, mph1Δ, tetO, Fta3-GFP
TetR-B1-Δk-RFP Fta3-GFP
TetR-B1-Δk-RFP
Fta3-GFP
mitotic cell
Fta3-GFP kinetochore marker
tethering Bub1 on TetO array
interphase cell
Fta3-GFP clustered kinetochores
tethering Bub1 on TetO array

Figure S3. Bypass of kinetochores in fission yeast: Mph1^{Mps1}-Bub1 SynCheck is independent from Spc7^{KNL1} and Bub3. Related to Figure 3

A) Co-immunoprecipitation (anti-FLAG) and immunoblots (anti-Bub1 and anti-Mad1) demonstrate that a Bub1-Mad1 complex is formed only in the arrested strains (expressing Bub1-full length or bub1-Δkinase). When Mph1 expression is inhibited (with the addition of thiamine to the media 15μM), the cells are not arrested and no Bub1-Mad1 complex is formed.

B) Images demonstrating that co-tethering of TetR-Bub1Δkinase with TetR-Mph1(303-678) in *bub3Δ* or *spc7-12TA* strains generates a robust mitotic arrest with short metaphase spindles. Scale bar, 10 microns.

C) Quantitation of arrested cells after 12, 14, 16 hrs of TetR-Bub1Δkinase with TetR-Mph1(303-678) induction in *Spc7+* and *spc7-12TA* strains. This experiment was repeated at least three times and data plotted as the mean ±SD.

D) Quantitation of arrested cells after 12, 14, 16 hrs of TetR-Bub1Δkinase with TetR-Mph1(303-678) induction in *Bub3+* and *bub3Δ* strains. This experiment was repeated 3 times and data plotted as the mean ±SD.

E) TetR-Bub1Δkinase-RFP forms red foci that did not co-localise with the kinetochore marker Fta3-GFP. Mitotic cells exhibit kinetochore localisation of Fta3-GFP, presented with 3 spots (for the 3 chromosomes). Scale bar is 10 microns. Schematic diagram of interphase and mitotic cells.

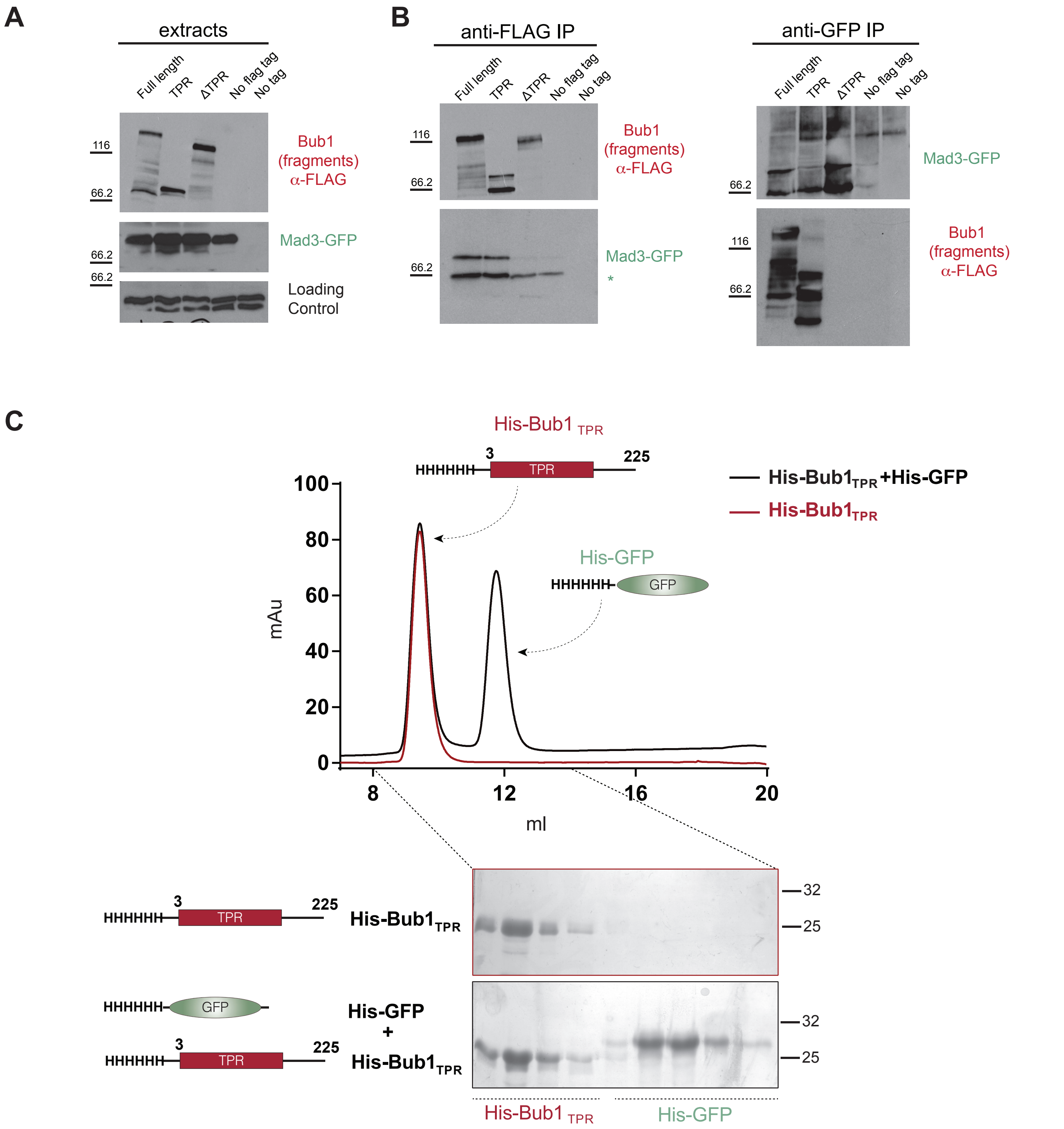

Figure S4. Fragments of Bub1 containing its TPR domain co-immunoprecipitate with Mad3. Related to Figure 4

A) Whole cell extracts were immunoblotted for Bub1 (anti-Flag) and Mad3 (anti-GFP). The loading control used here was tubulin.

B) Mad3-GFP co-immunoprecipitated only with TetR-Bub1 fragments that contain a TPR domain. Both anti-FLAG and anti-GFP immunoprecipitations were performed. TetR-Bub1 fragments were detected with anti-Flag antibody and the associated Mad3 with anti-GFP antibodies.
* a cleaved form of Mad3-GFP bound non-specifically to the anti-FLAG beads.

C) Recombinant Bub1-TPR does not bind GFP. SEC profiles and respective SDS–PAGE analysis of: His-Bub1TPR elutes at 9.5mls, His-GFP elutes at 11.8mls and His-Bub1TPR/His-GFP mix elutes at the same places as there is no Bub1TPR- GFP complex formation. All samples were injected into a Superdex 75 increase 10/300. Absorption at 280 nm (mAU, left y-axis) is plotted against elution volume (ml, x-axis).
